# Advances in Nanohybrid Membranes for Dye Reduction: A Comprehensive Review

**DOI:** 10.1002/gch2.202300052

**Published:** 2023-12-21

**Authors:** Mahsa Taheri

**Affiliations:** ^1^ Civil and Environmental Engineering Department Amirkabir University of Technology (AUT) Hafez Ave. Tehran 15875‐4413 Iran

**Keywords:** membrane, nanohybrid, dyes removal, treatment, wastewater

## Abstract

Separating valuable materials such as dyes from wastewater using membranes and returning them to the production line is a desirable environmental and economical procedure. However, sometimes, besides filtration, adsorption, and separation processes, pollutant destruction also can be suitable using photocatalytic membranes. The art of producing nanohybrid materials in contrast with nanocomposites encompasses nanomaterial synthesis as a new product with different properties from raw materials for nanohybrids versus the composition of nanomaterials for nanocomposites. According to the findings of this research, confirming proper synthesis of nanohybrid is one challenge that can be overcome by different analyses, other researchers' reports, and the theoretical assessment of physical or chemical reactions. The application of organic‐inorganic nanomaterials and frameworks is another challenge that is discussed in the present work. According to the findings, Nanohybrid Membranes (NHMs) can achieve 100% decolorization, but cannot eliminate salts and dyes, although the removal efficiency is notable for some salts, especially divalent salts. Hydrophilicity, antifouling properties, flux, pressure, costs, usage frequency, and mechanical, chemical, and thermal stabilities of NHMs should be considered.

## Graphical abstract

Nanohybrid membranes (NHMs) can be divided to two categories of photocatalytic and non‐photocatalysts. Hence, approximately 50% of NHMs are produced by Phase Inversion (PI) and most of NHMs are polymeric, the current paper will categorize NHMs according to these criteria.

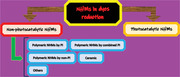

## 1 Introduction

The textile industry by ≈8 million.m^3^ d^−1^ wastewater is the fourth most important industry in terms of quantity in industrial wastewater discharge after the petrochemical, paper, and mining industries.^[^ ^1^ ^]^ The textile dyes industry and its impacts on the environment have been the focus of researchers for numerous years.^[^ ^2^, ^3^, ^4^ ^]^ The usual weak bind of textile dyes to fabrics causes severe water pollution when discharged. There are various methods for textile dye removal.^[^ ^5^, ^6^, ^7^, ^8^ ^]^ But in recent years, researchers have emphasized dye recovery and reuse in the same industry as a perfect solution.^[^ ^9^ ^]^ Although other treatment processes can be applied to help the process, many studies have opted for membrane separation as an inseparable part of the process within the textile industry.

Membranes can be applied in the membrane bioreactor (MBR), photocatalytic membrane reactor (PMR), and separation processes such as Nano‐filtration or Reverse Osmosis (RO). Membranes are also used in fuel cells or Microbial Fuel Cells (MFCs) to produce chemical energy.^[^ ^10^ ^]^ Membranes in MBR systems are usually used in the form of ultra‐ and microfiltration membranes.^[^ ^11^ ^]^ Scientists have acquired momentum recently in nanomaterials (e.g., nano devices/membranes).^[^ ^12^ ^]^ The first human‐fabricated nanodevice was a gold‐silver Lycurgus Cup in the fourth century.^[^ ^13^ ^]^

Nanoparticles are found in 1) human bodies (e.g. Antibody and DNA), 2) pathogens or living/nonliving organisms that exchange genetic information directly with living organisms (virus), and 3) in nature (e.g. gold and silver).^[^ ^13^, ^14^, ^15^ ^]^ Nanomaterials are defined as at least one dimension <100 nm or with internal structures measuring 100 nm or less.^[^ ^16^ ^]^ Nanoparticles or nanomaterials can be classified according to different criteria such as dimension, material, and manufacturing,^[^ ^17^ ^]^ organic/inorganic,^[^ ^18^ ^]^ and engineered/natural,^[^ ^19^ ^]^ respectively. At least 10^12^ g of natural nanomaterials move around the earth annually.^[^ ^19^ ^]^ Nanoparticles can be produced by different methods, such as sol–gel and Precipitation Polymerization (PP).

A nanocomposite is a composite material with features measured in nanometers.^[^ ^20^ ^]^ Nanocomposites are composite materials that contain at least one component of the nanometric scale.^[^ ^21^ ^]^ In polymer nanocomposites, the polymer and nanomaterial components usually act as a matrix (substrate) and nanofiller, respectively.^[^ ^22^ ^]^ As an example, nanocomposite films can be produced by mixing 3 wt.% of the specific jute nanofibrils.^[^ ^22^, ^23^, ^24^ ^]^ The word fillers in nanocomposites are usually reminiscent of the nanoparticles, but it can also be applied to nanofibrils (nanofibers).^[^ ^25^, ^26^ ^]^ The amount of fillers can be determined by formulas, such as those related to the Cox–Krenchel theory. These formulas determine the mechanical and thermal properties of the nanocomposites.^[^ ^25^, ^27^ ^]^ Abbas et al.^[^ ^28^ ^]^ defined nanocomposites as nanoparticle‐reinforced materials usually containing up to 5 wt.% nanoparticles.

**Figure 1:**
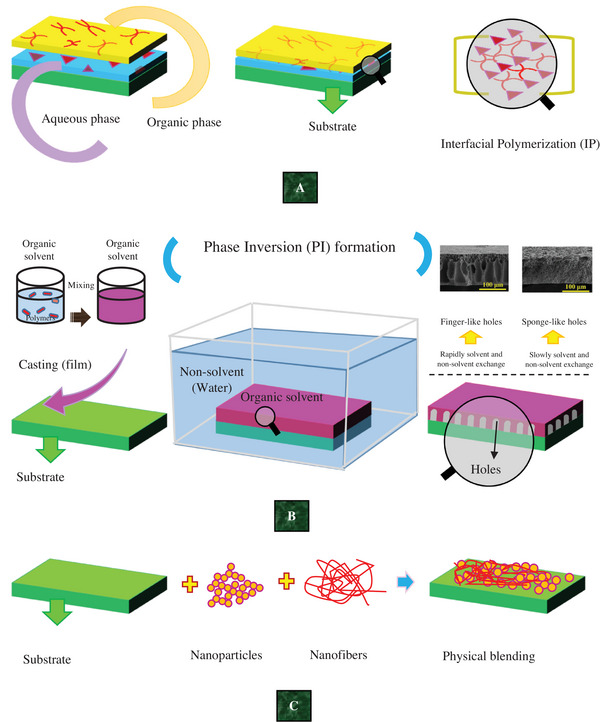
Schematic figures of A) IP,^[^ ^34^ ^]^ B) PI^[^ ^35^ ^]^ (Real photos of finger‐like and sponge‐like reprinted with permission from Chen et al.^[^ ^36^ ^]^ © 2020 American Chemical Society.), and C) Physical mixing/ blending as possible methods in NHM production.^[^ ^10^ ^]^

Composite membranes for dye removal by Nanofiltration (NF) can be manufactured using controllable glycerol co‐coating versus traditional coating, in which glycerol is a simple and environmentally friendly additive.^[^ ^29^ ^]^ Nanocomposite Membranes (NCMs) are obtained by incorporating organic or inorganic nanoparticles into a polymeric membrane matrix.^[^ ^30^ ^]^ A highly porous substrate coated with a dense film of a different polymer called a Thin Film Composite (TFC) membrane is usually used in water purification/desalination.^[^ ^31^ ^]^ Thin Film Nanocomposite (TFN) Membranes are a modification of the existing TFCs prepared through methods such as Interfacial Polymerization (IP), Phase Inversion (PI), mixing (solution mixing), and polymer melt blending.^[^ ^32^, ^33^ ^]^ Among TFC production processes, IP is the most common process.^[^ ^33^ ^]^ **Figure** **1** depicts IP, PI, and mixing processes. Solution mixing involves mixing materials such as nanoclay and organo‐liquid/organic solvent to disperse nanomaterials and separate stacks of sheets for any purpose, including manufacturing the membrane.^[^ ^37^ ^]^ Polymer melt blending as a method for NHM production is like mixing (as shown in Figure 1), but during this process, polymer melting, blending with nanomaterials, extrusion, and cooling of the nano‐polymer are performed.^[^ ^38^ ^]^

**Figure 2:**
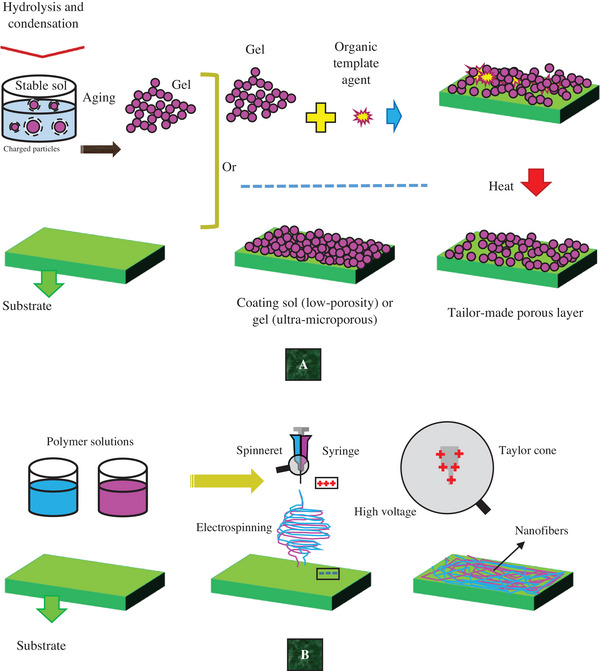
A) Sol–gel^[^ ^39^, ^40^, ^44^ ^]^ and B) electrospun processes as possible methods in NHM production.^[^ ^45^, ^46^ ^]^

The word nanohybrid was coined in 1996.^[^ ^39^, ^40^, ^41^, ^42^ ^]^ Nanohybrid materials are single synthetic materials linked by non‐covalent (hydrogen bond, van der Waals force, or electrostatic force) or covalent bonds that result in molecular nanomaterial or nanoscale dimensions, with different properties from the initial materials.^[^ ^43^ ^]^ Nanohybrid membranes (NHMs) were initially produced from sol–gel processes some 25 years ago according to **Figure** **2**A.^[^ ^39^, ^40^, ^44^ ^]^ The first articles related to producing NHMs using polymer electrolyte membrane fuel cells were published ≈20 years ago.^[^ ^47^ ^]^ The electrospun process (Figure 2B) is another method for fabricating membranes and NHMs. Nanofibers produced during electro‐spinning became famous in the late 20th century.^[^ ^48^ ^]^

In water or wastewater treatment, containing nanosilver particles and a support layer containing multi‐walled carbon nanotubes (MWNTs).^[^ ^49^ ^]^ The nanoparticles of Ag and MWNTs were distributed in the thin‐film layer and substrate, respectively. Spectroscopic characterization revealed that the nanosilver carbide nanoparticles have adhered to the components of the thin film layer and have acted as a modifier for the MWNT substrate layer.^[^ ^49^ ^]^

Although using NHMs cannot guarantee better performance, anti‐fouling properties, and lifetime,^[^ ^50^ ^]^ they can solve the problems of conventional membranes.^[^ ^10^, ^51^ ^]^ NHMs have some limitations and challenges, including 1) Ineffective in reducing small size ions such as angstrom‐sized ions from solution (Sodium chloride) and even dyes (Reactive Orange 16), that salts can be found easily in the dye‐containing wastewaters,^[^ ^52^, ^53^, ^54^ ^]^ 2) nanoparticle aggregation as one of the main challenges in production and leaching nanomaterials especially nanoparticles as a common health problem,^[^ ^55^ ^]^ 3) stability of the membrane or thermal stability of nanofibers,^[^ ^55^, ^56^, ^57^ ^]^ 4) Expensive especially when raw nanomaterials are not cheap,^[^ ^10^ ^]^ 5) High technique may be required to manufacture NHMs.^[^ ^58^ ^]^

NHMs have received attention for dyes reduction from the early last decade.^[^ ^59^, ^60^, ^61^ ^]^ Nanohybrid‐based membranes in water and wastewater treatment are rarely discussed in textbooks.^[^ ^62^, ^63^ ^]^ In addition, scientific papers have seldom reviewed NHMs in fuel cells and photocatalytic applications.^[^ ^10^, ^64^, ^65^, ^66^ ^]^ Therefore, this paper aims to discuss nanohybrid‐based membranes for dye reduction.

## 2 Nanohybrid Membranes in Dyes Reduction

### 2.1 Categories and General Principles

**Figure 3:**
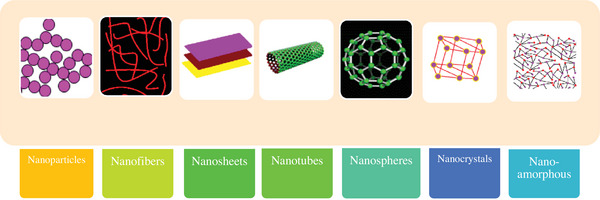
Nanomaterial classification according to the most famous molecular shapes.^[^ ^69^, ^70^ ^]^

Nanoparticles can be divided into carbon‐based, organic, and inorganic particles.^[^ ^67^ ^]^ **Figure** **3** presents the familiar forms and categorization of nanomaterials. However, there is an additional grouping that cannot be represented based on shape alone, which is the distinction between manufactured and naturally occurring nanomaterials.^[^ ^68^, ^69^, ^70^ ^]^ Nanomaterial classification according to the dimensions of 1) 0 D (fullerene), 2) 1 D (CNTs), 3) 2 D (graphene), and 4) 3 D (graphite) is also depicted.^[^ ^71^ ^]^ As mentioned in the introduction, PP was a nanoparticle production method. PP easily produces different uniform polymer particles with no surfactant.^[^ ^72^ ^]^ Distillation Precipitation Polymerization (DPP) is a process for controlling the formation of microspheres by distilling solvents via polymerization.^[^ ^72^ ^]^ DPP is a rapid novel polymerization method for preparing monodispersed micro/nanoparticles with remaining notable vinyl groups on the surface.^[^ ^73^ ^]^

Agglomeration, and dispersion can describe nanomaterials. In another classification, single, nanocomposite, and nanohybrid categories can be mentioned.^[^ ^68^ ^]^ According to the molecular structure and properties, the application of nanomaterials is different. Carbon‐based nanocomposites as one of the most famous groups of nanomaterials can act in electrocatalysis, photocatalysis, piezocatalysis, adsorption, and separation via membrane processes.^[^ ^74^, ^75^, ^76^ ^]^

**Figure 4:**
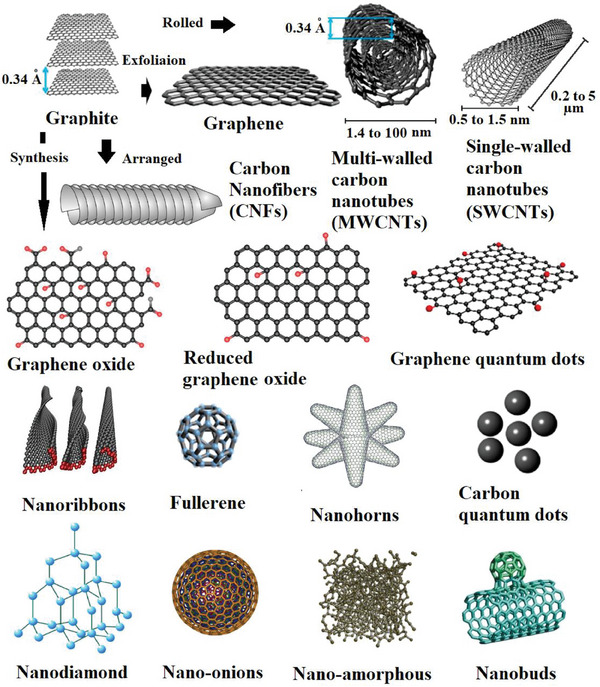
Nanocarbon materials.^[^ ^77^, ^78^, ^82^, ^83^, ^85^, ^87^, ^88^, ^89^, ^90^, ^91^, ^92^, ^93^, ^94^, ^95^ ^]^ Reprinted with permission. Reprinted Nanoribbons with permission from Kit et al.^[^ ^96^ ^]^ (2012) by the American Physical Society.

Carbon‐based nanomaterials comprise fullerene, nanohorns, nano‐amorphous, nanobuds, nano‐onions, nanoribbons, nanodiamonds, graphite, graphene, Graphene Oxide (GO), Reduced Graphene Oxide (RGO), Carbon Quantum Dots (CQDs), graphene quantum dots (GQDs), Carbon Nanofibers (CNFs), Carbon Nanotubes (CNTs) including Single‐Walled Carbon Nanotubes (SWCNTs) and MWCNTs.^[^ ^77^, ^78^, ^79^, ^80^, ^81^, ^82^, ^83^, ^84^, ^85^, ^86^ ^]^ **Figure** **4** shows the nanocarbon materials. Other carbon nanohybrids that are like nanobuds are not presented in the figure. CNFs are described in some references as cup‐stacked and stacked‐cup carbon nanofiber helical structures.^[^ ^97^ ^]^

Nanocarbon materials and quantum dots (e.g., CdSe and ZnS) can be classified as organic and inorganic groups, respectively.^[^ ^98^ ^]^ Polystyrene, dendrimer, liposomes, and ferritin are examples of organic nanoparticles.^[^ ^99^ ^]^ Some of the most important non‐metallic and metallic (inorganic) nanoparticles include Au, Ag, Ti, Al, Zn, Zr, Si, and some of their combinations, especially with oxygen.^[^ ^100^ ^]^ Though, other studies mentioned Cu, Fe_3_O_4_, Nickel Oxide (NiO), Zinc Oxide (ZnO), CeO_2_, Titanium Dioxide (TiO_2_), Fe_2_O_3_, FeO(OH), CuO, MgO, Silicon Dioxide (SiO_2_), Yttrium oxide (Y_2_O_3_) as metal‐based (inorganic) nanomaterials, which can be obtained through metal reduction, hydrothermal/solvothermal, sonochemical, sol–gel, combustion, chemical, and vapor deposition.^[^ ^68^, ^98^, ^99^ ^]^

**Figure 5:**
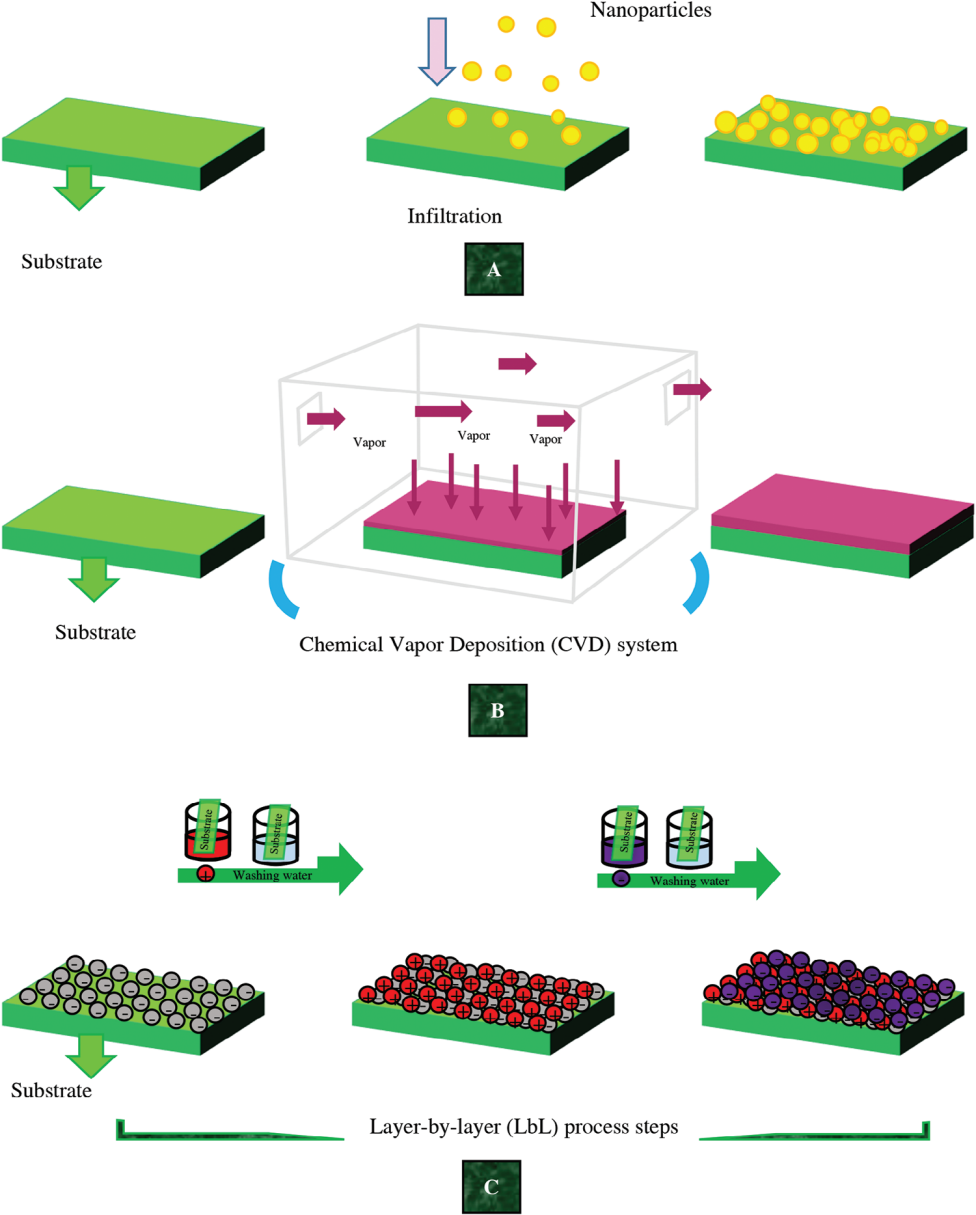
A) Infiltration,^[^ ^10^ ^]^ B) CVD,^[^ ^102^ ^]^ and C) LbL processes as possible methods^[^ ^101^ ^]^ in NHM production.

Ag, Au, and Cu nanomaterials can be obtained by metal reduction or chemical and vapor deposition.^[^ ^68^ ^]^ Chemical Vapor Deposition (CVD) is also one of the methods of membrane manufacturing. Infiltration, CVD, and Layer‐by‐layer (LbL) processes as possible methods for NHM production are depicted in **Figure** **5**.^[^ ^101^ ^]^ Three generations of nanomaterial: 1) graphene, CNTs, and fullerene, 2) doped CNTs and graphene, and 3) nanohybrids like nanobuds (Figure 4) as carbon–carbon nanohybrids or Boron Nitride Nanotubes (BNNTs) as carbon‐noncarbon nanohybrids was reported.^[^ ^103^ ^]^

**Figure 6:**
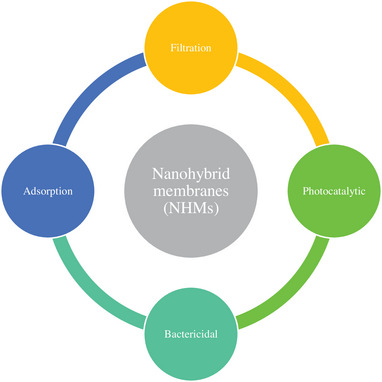
Possible characteristics of NHMs related to pollution reduction.^[^ ^106^ ^]^

NHMs are applied due to gas separation,^[^ ^39^, ^40^, ^44^ ^]^ fuel cell,^[^ ^10^ ^]^ liquid separation/filtration,^[^ ^49^ ^]^ pollution adsorption,^[^ ^104^ ^]^ photocatalytic,^[^ ^105^ ^]^ and anti‐microbial properties.^[^ ^49^ ^]^ **Figure** **6** shows the characteristics of NHMs related to pollution reduction.^[^ ^106^ ^]^ Three main groups of 1) polymeric, 2) ceramic, and 3) novel materials, including graphene, Vertically Aligned Carbon Nanotubes (VACNTs), TiO_2_ nanotubes, and their combinations can be mentioned for NHMs.^[^ ^63^ ^]^ **Table** **1** shows nanomaterial properties in membrane manufacturing including stability, antifouling, and water permeability for some of the most important nanomaterial. Graphene, GO, and their combinations are widely used in membrane production.^[^ ^100^ ^]^

**Table 1:** Nanomaterial properties in membrane manufacturing.^[^ ^59^, ^63^, ^107^, ^108^, ^109^, ^110^, ^111^ ^]^

Nanomaterial Stability				Nanomaterial Fouling and Permeability	
Thermal Stability^a)^	Mechanical Stability	Not Leaching	Proper Distribution (Not Agglomerate)	Antifouling	Permeability
Metal/Metal Oxide^b)^ CNTs Graphene^c)^ Fullerene^c)^ Nanoclay^d)^	Metal/Metal Oxide CNTs Graphene^e)^ Fullerene^f)^ Nanoclay Nanofiber^g)^	CNTs^h)^ Graphene^i)^ Fullerene^j)^ Nanoclay^k)^	Metal/Metal Oxide^l)^ Graphene^m)^ Fullerene^n)^ Nanoclay^o)^ Nanofiber^p)^	Metal/Metal Oxide CNTs Graphene Fullerene Nanoclay Nanofiber	Metal/Metal Oxide^q)^ CNTs Graphene^r)^ Nanoclay

^a)^ Thermogravimetric Analysis (TGA);

^b)^ Alharbi et al.^[^ ^112^ ^]^ evaluated metal/metal oxide;

^c)^ Weight loss of 50% (up to 600°C) for GO^[^ ^113^ ^]^ and fullerene.^[^ ^114^ ^]^ Weight loss of 20% (up to 600 °C) for graphene^[^ ^115^ ^]^ and no weight loss for graphite.^[^ ^115^ ^]^ Just 20% weight loss (up to 600 °C) for RGO;^[^ ^116^ ^]^

^d)^ Weight loss of 20–30% up to 600 °C;^[^ ^117^ ^]^

^e)^ A proper mechanical substrate lets graphene endure pressures ten times more than Reverse Osmosis in seawater desalination;^[^ ^118^ ^]^

^f)^ C60 and C70 are best;^[^ ^119^ ^]^

^g)^ Tensile strengths;

^h)^ CNTs do not cause antibacterial properties;^[^ ^120^ ^]^

^i)^ GO sheets anchor nanoparticles^[^ ^121^ ^]^ and GQDs did not show leaching;^[^ ^122^ ^]^

^j)^ Fullerenes are leached out because of the high density of wall defects;^[^ ^123^ ^]^

^k)^ Leaching of nanoclay will be minimized using it combined with other effective materials such as polymers;^[^ ^124^ ^]^

^l)^ Proper distributions.^[^ ^125^ ^]^ Agglomeration occurs when metal or metal‐oxide are added directly to the polymer solution.^[^ ^126^, ^127^ ^]^ CNTs are also hydrophilic and form agglomerates in polymers;^[^ ^128^ ^]^

^m)^ Narrow distribution in size;^[^ ^129^ ^]^

^n)^ Lateral distribution is depicted;^[^ ^130^ ^]^

^o)^ By dispersing of nanoclay in the membrane's matrix;^[^ ^131^ ^]^

^p)^ Agglomeration of CNF as a nanofiber was increased by increasing CNF from 0.5 to 1.5 wt.%;^[^ ^132^ ^]^

^q)^ Hydrophilic materials increase the water permeability of a membrane.^[^ ^133^ ^]^ Metal oxides sometimes decrease the water permeability of the membranes because of high doping;^[^ ^134^ ^]^

^r)^ Peng et al.^[^ ^135^ ^]^ fabricated nanohybrid RGO@SiO_2_ NPs to overcome low flux and the problem of easily damaged GO‐based membranes.

### 2.2 Non‐Photocatalytic NHMs

#### 2.2.1 Polymeric NHMs Produced by PI

**Figure 7:**
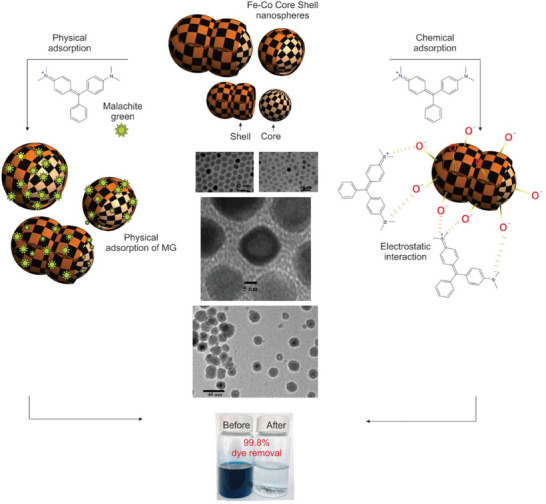
All the illustrations except real white–black images in the center: Iron‐cobalt (Fe‐Co) core‐shell nanospheres adsorb Malachite Green dye.^[^ ^136^ ^]^ Reprinted with permission. Transmission Electron Microscopy (TEM) as four white–black images in the center: Fe‐Co nanoparticles with a scale bar of 40 nm (top left), Fe‐Co with an average 10 nm diameter and 20 nm scale bar (top right), Fe‐Co nanoparticles with an oxide shell when exposed to air, the scale bar is 5 nm (middle), Fe‐Co with average 20 nm diameter after 30 min annealing at 500 ^o^C, the scale bar is 40 nm (bottom). Reprinted with permission from Chaubey et al.^[^ ^139^ ^]^ 2007 American Chemical Society.

The summary of researchers’ findings for non‐photocatalytic polymeric NHMs produced by PI for dye reduction and the selected figure from the cited papers (**Figure** **7**) are presented in **Table** **2**. For better illustration, in Figure 7, the central part was added to the images.

**Table 2:** Summary of findings for non‐photocatalytic polymeric NHMs produced by PI for dye reduction.

No.	Reference and Figure	Membrane Material	Type of Pollution	Type of membrane	Analysis	Main Findings	Maximum Flux
1	[53]	QSiPD from PDA using PES	Congo Red, NaCl, CaCl_2_, MgSO_4_, Na_2_SO_4_	NF/UF, Not mentioned	SEM, EDX, XPS, FTIR, Zeta potential, TGA, Raman spectra, contact angle, porosity, tensile stress, number of bacteria, flux, and rejection	Rejections: Congo Red (>99.9%), Na_2_SO_4_ (67%) > NaCl (47%) > MgSO_4_ (46%) > CaCl_2_ (45%)	5590 l/(m^2^h.MPa)
2	[136] Figure 7	Fe‐Co and Polyvinylidene fluoride (PVDF)	Malachite Green	MF, Not mentioned	XPS, TEM, XRD, SEM, FTIR, BET, Fe‐Co loading, contact angle, dye concentration, flux and rejection, flux and rejection recovery ratio, fouling and resistances, leaching tests	Malachite Green (97%) was achieved through the M‐0.1 membrane (containing 0.1% Fe‐Co loading), hydrophobicity increased by adding nanomaterials but had a peak.	42946 l/(m^2^h.MPa)^a)^
3	[137]	Lemon‐derived CQDs‐ Silver nanoparticles (Ag/CQDs) and PSF	Tartrazine	UF, Not mentioned	SEM, EDX, FTIR, XRD, Zeta potential, five different loading of Ag/CQD, reuse cycle, contact angle, pore size, porosity, the viscosity of the membrane casting solution	Excellent pure water permeability (169%) and dye rejection (92%), lower flux decline, higher reversible and lower irreversible fouling.	647.5 l/(m^2^h.MPa)
4	[138]	ZnO and TiO_2_ nanoparticles as ZnO/TiO_2_/PSF	Congo Red	UF^b)^, Not mentioned	SEM, EDX, porosity, flux, antifouling, roughness, contact angle, mechanical stability (tensile stress and break elongation)	Antifouling and hydrophobicity properties, increased permeability (254%), tensile (3 MPa), and elongation (1%) stress increased, nanoparticle distribution, retention of 50–80% for Congo Red dye from control membrane to NHM	300–600 l/(m^2^h.MPa)

^a)^ Calculated;

^b)^ Estimated.

Some abbreviations in Table 2 are Quaternized Polydopamine (QSiPD), Polydopamine (PDA), Polyethersulfone (PES), Polyvinylidene fluoride (PVDF), and Polysulfone (PSF). The columns related to the type of membrane and analysis in Table 2 also include other abbreviations: Ultrafiltration (UF), Microfiltration (MF), Field Emission‐Scanning Electron Microscopy (FE‐SEM), Energy Dispersive X‐Ray Analysis (EDX), X‐Ray Diffraction Analysis (XRD), X‐Ray Photoelectron Spectroscopy (XPS), Brunauer–Emmett–Teller (BET), and Atomic Force Microscopy (AFM). In some references, the dynamic water contact angle is applied instead of the contact angle. In addition, the term Non‐Solvent Induced Phase Separation (NIPS) in some references is another name for PI.

The production of QSiPD‐PES NHM is shown in Ismail et al.^[^ ^53^ ^]^ The production of QSiPD‐PES NHM process comprises: 1) QSiPD nanohybrid from hydrolyzed QSiP (QSiP‐OH) and self‐polymerized PDA via condensation in a slightly basic (pH 8.5) medium using PES, and 2) QSiPD‐PES NHM manufacturing in the research.^[^ ^53^ ^]^ The results of XPS for investigating the formation of crosslinked Si–O–Si linkage with the membrane confirmed QSiP as a layer on the top surface of the changed PDA (QSiPD). The removal of 1 g L^−1^ monovalent (NaCl) and divalent (CaCl_2_, MgSO_4_, Na_2_SO_4_) salt solutions and 10 mg L^−1^ Congo Red was examined.^[^ ^53^ ^]^ The number of attached bacterial cells on the membrane surface was also evaluated to show the bacteria‐killing capability of NHMs. In addition, strong antibacterial activity was reported against Escherichia coli (*E. coli*).^[^ ^53^ ^]^

The Zeta potential of QSiPD was 11.26 mV in comparison with −31.93 mV for PDA.^[^ ^53^ ^]^ At approximately neutral pH, phenolic groups' deprotonation caused a negative surface charge of the PDA.^[^ ^53^ ^]^ The positive charge of the QSiPD nanohybrid is because of quaternary ammonium.^[^ ^53^ ^]^ The surface charge of a membrane as zeta potential, especially for UF and NF, plays an important role in the solute rejection. Most polymeric membranes in the wide ranges of pH's wastewater exhibit negative charge, and therefore, repel compounds with negative charges and divalent anions. The membrane charge can be changed by surface reformation to reject specific solutes.^[^ ^140^ ^]^

Table S1 (Supporting Information) shows the non‐photocatalytic nanohybrid based membrane (NHM) categories for dye reduction. According to Table 2 and Table S1 (Supporting Information), the QSiPD‐PES NHM is a non‐carbon‐based membrane produced by PI. Table 2 shows that carbon‐based materials as the most famous group of NHMs cannot promise better performance. In addition, TGA analysis shows acceptable thermal stability of QSiPD as a new material in NHM fabrication for dye removal because of Si–O–Si linkages. According to Table 2, SEM followed by EDX are inseparable parts of NHM production in the studies. The rejection rate of QSiPD‐PES NHM is high compared to the other non‐photocatalytic polymeric NHMs produced by PI in Table 2. In addition, the maximum flux of the QSiPD membrane is notable among NF or UF membranes in Table 2.

As depicted in Figure 7, the adsorption of 20 to 400 mg L^−1^ Malachite Green on the nanomaterial was examined, but the initial dye concentrations were limited to 50 to 100 mg L^−1^ for the dye separation via NHMs.^[^ ^136^ ^]^ In addition, XPS, TEM, and XRD tests were only performed on nanomaterial. Leaching tests were also accomplished and the results show that no leaching of Fe or toxic Co occurred. The MF polymeric membrane comprising Fe‐Co shows high flux during the experiments among other membranes in Table 2. Flux of 214.73 l/(m^2^h) in 0.005 MPa was reported that convert to MPa in Table 2 for better comparison.^[^ ^136^ ^]^ Lemon‐derived CQDs‐Silver (Ag/CQDs) nanoparticles were used as the membrane nanofiller for 10 mg L^−1^ Tartrazine filtration.^[^ ^137^ ^]^ The modified membrane exhibited higher reversible fouling.^[^ ^137^ ^]^ The membrane pores were larger than Ag/CQDs nanoparticles; therefore, in this case, adding nanomaterials caused better filtration by providing a less dense membrane. Although contrary to,^[^ ^141^ ^]^ all the membranes in Table 2 have a denser volume after adding nanoparticles according to the SEM images.

The final membrane type did not mention in the research,^[^ ^138^ ^]^ but the initial membrane was UF, and reduced pore size in UF was reported after adding nano. Congo Red concentration was 100 mg L^−1^, which is an intermediate concentration. Membranes' inorganic nanoparticles play important roles, particularly in hydrophobicity/hydrophilicity modification of the active surface membrane. ZnO/TiO_2_/PSf membrane caused an increase in permeability (254%) with 65% retention (higher fluxes) in comparison with the PSf‐control membrane. ZnO/TiO_2_ was built with three concentrations (especially 1 and 0.5 wt.%), two of which presented antifouling properties compared to the control membrane. Adding two types of nanoparticles (ZnO and TiO_2_) led to better nanoparticle distribution in the membrane matrix because of repulsive interactions between them, and therefore, clearly improved NHMs.^[^ ^138^ ^]^ As mentioned previously with nanoparticles, appropriate distribution of nanomaterials is a major concern that is discussed in this research.^[^ ^138^ ^]^ As seen in Table 2, most of the factors discussed in this study are related to good distribution.

The following conclusions can be made for non‐photocatalytic polymeric NHMs produced by PI for dyes reduction:

- None of the NHMs were made of GO or related materials, although carbon‐based NHMs were reported.
- MF to UF were produced in this section.
- The dye removed over 80% in all cases.
- The single dye, with salts in one case, was removed in this section.
- The charge of dyes was positive or negative, one of them was azo and the other was triarylmethane.
- The membrane was unsuccessful in the salt rejection.
- The addition of nanometals increased hydrophobicity, but it may reach a peak after which hydrophobicity may decrease despite the continued addition of nanometals.
- The good distribution of nanomaterials was reported in this section.
- Adding nanomaterials can create larger pores instead of smaller pores.
- Leaching tests were also accomplished, especially for toxic nanomaterial.

#### 2.2.2 Polymeric NHMs Produced by Combined PI

**Table** **3** presents a summary of the findings of researchers for non‐photocatalytic polymeric NHMs produced by PI in combination with other processes for dye reduction. Some abbreviations in Table 3 are Silicon Nitride (Si_3_N_4_), Polyacrylonitrile (PAN), Dynamic Light Scattering (DLS), Attenuated Total Reflectance‐Fourier Transform Infrared Spectroscopy (ATR‐FTIR), and Total Organic Carbon (TOC).

**Table 3:** Summary of findings for non‐photocatalytic polymeric NHMs produced by the PI method combined with other processes for dye reduction.

No.	Reference and Year	Membrane Materials	Type of Pollution	Type of Membrane	Method	Analysis	Main Findings	Maximum Flux
5	[141]	GO‐based TiO_2_ nanomaterials (TiO_2_@RGO) into the TFN. The membrane comprises PAN, Si_3_N_4_, and PA	Rose Bengal	NF, Hollow fiber	Functionalized, PI, and IP	FE‐SEM, AFM, XRD, TEM, FTIR, DLS, contact angle, flux, permeability, and antifouling	Solvent channeling (GO), hydrophilicity (amino‐functionalized and TiO_2_), antifouling properties, structural stability, suitable for polar solvent (ethanol and isopropanol).	20–100 l/(m^2^h.MPa) for solvents
6	[54]	Zwitterionic GO, polyvinylpyrrolidone (PVP), and PSF	Reactive Black 5, Reactive Orange 16, Protein	UF, Hollow fiber	DPP and PI	FTIR, XRD, TGA, EDX, FE‐SEM, XPS, TEM, zeta potential, contact angle, pH, flux, pressure, antifouling, resistances, salt and salt/dye mixture	Reactive Black 5 (99%) and Reactive Orange 16 (74%) rejection. Complete NaCl permeation and Na_2_SO_4_ less rejection (<5%).	496 l/(m^2^h.MPa)
7	[142]	ZnO nanoparticles‐ Carboxylated Graphene Oxide nanosheets (ZnO/CGO) and PES	Methylene Blue, Rhodamine B	NF, Hollow fiber	Spinning, Physical mixing/ blending, and PI	TEM, XRD, ATR‐FTIR, XPS, SEM, EDX, Zeta potential, TGA, AFM, contact angle, stability, permeability, antifouling, pore size, and porosity	Methylene Blue: 98.6% and Rhodamine B: 98.5%. better hydrophilicity, antifouling, stability, mechanical strength, and surface roughness.	2689 l/(m^2^h.MPa)
8	[135]	SiO_2_ nanoparticles in PVDF/RGO@SiO_2_/PDA	Methylene Blue, Sodium Dodecyl Sulfate (SDS)/diesel oil/H_2_O emulsion	Not mentioned, Not mentioned	PI, Deposition, and Filtration	SEM, AFM, XPS, ATR‐FTIR, TEM, contact angle, TOC, antifouling, contact angle, flux, pH	Low water flux GO‐based membrane using SiO_2_ between graphene layers, successful oil‐water emulsion and dye wastewater treatment, Dopamine (DA) increased hydrophilicity.	4755 l/(m^2^h.MPa)
9	[143]	GO and Lithium Chloride (LiCl) blending PVDF (PVDF/GO/LiCl)	Rhodamine B	UF, Not mentioned	Precipitation, and PI	XRD, Zeta potential, SEM, AFM, FTIR‐ATR, XRD, TEM, dosage, contact angle, pore size, flux, antifouling	Increasing flux, improvement of hydrophilicity, dye removal rates, and flux recovery ratios were over 80% and 78.2%, respectively.	484‐619 l/(m^2^h.MPa)

Both materials and processes applied in Table 3 are more complicated than those in Table 2. For example, a comparison between No. 5 in Table 3 and No. 4 in Table 2 shows the more complicated results in Table 2. The type of membrane is the other notable note in Table 3. Hollow fiber is mentioned for three of the rows in Table 3. In addition, all the membranes in Table 3 have lower fluxes than the fluxes of the membranes in Table 2 and MF is not mentioned as an existing type of membrane in Table 3. The application of the NHMs in Table 3 is also more extensive; the membranes separate more complicated wastewater than the wastewater mentioned in Table 2.

High concentrations of 500 mg L^−1^ Rose Bengal were removed by incorporating nanomaterials in the substrate.^[^ ^141^ ^]^ The researchers illustrated TiO_2_@RGO production and incorporation into the TFN, the substrate formation of PAN on a Si_3_N_4_ ceramic membrane, and PA produced through the IP process of 1,3,5‐benzenetricarbo nyltrichloride (TMC) and m‐phenylenediamine (m‐PDA) monomers. XRD, TEM, FTIR, and DLS analyses were accomplished on nano. The GO nanosheets structure helps organic solvent channeling and TiO_2_ has super hydrophilic characteristics; therefore, the highly permeable hydrophilic amino‐functionalized membrane was produced with antifouling properties and structural stability. Microwave irradiation was the process for TiO_2_@RGO synthesis, by providing enough active sites on the decoration of TiO_2_ nanomaterials on the GO sheets. Hydrophilic NHMs are suitable for polar solvents such as ethanol and isopropanol while suppressing non‐polar solvents including n‐heptane and n‐hexane.^[^ ^141^ ^]^ The only membrane for solvents in Table 2 belongs to No. 5, which differentiates this research from other research that investigated water as a solvent. Also, the dye concentration in this study is the highest value between the values of Tables 2, 3, which has also been reported in the work of Iqbal et al.^[^ ^136^ ^]^ with a high dye circuit, albeit less than the present study, and the dye concentration in other studies has been significantly less than the two mentioned studies.

The concentration of the selected dyes by Syed Ibrahim et al.^[^ ^54^ ^]^ was as low as 10 mg L^−1^. The research demonstrates the production of GO@poly (SBMA‐co‐MBAAm) NHM using N, N′‐methylenebis (acrylamide) (MBAAm) and [2‐(Methacryloyloxy)ethyl]dimethyl‐(3‐sulfopropyl) ammonium hydroxide (SBMA) monomers before incorporation with PSF pristine membrane.^[^ ^54^ ^]^ DPP was applied for nanohybrid and PI for membrane manufacturing.^[^ ^54^ ^]^ The membrane revealed notable fouling alleviation with a high Flux Recovery Ratio (FRR) of 73% toward the famous Bovine Serum Albumin (BSA) protein.^[^ ^54^ ^]^ Although Syed Ibrahim et al.^[^ ^54^ ^]^ reported the use of NHM for the separation of salt/dye mixtures in wastewater, according to Table 3, NHM was not suitable for salt rejection.

Zhu et al.^[^ ^143^ ^]^ discussed non‐Electrospun fiber spinning,^[^ ^144^ ^]^ which is unique in this study. TEM, EDX, XRD, ATR‐FTIR, and XPS tests were accomplished with nano in the study.^[^ ^142^ ^]^ The dye concentrations were 50 mg L^−1^ Methylene Blue and 2 mg L^−1^ Rhodamine B. Methylene Blue (98.6%) and Rhodamine B (98.5%) rejections were reported in Table 3.^[^ ^142^ ^]^ The positive effects of surface functionality, mechanical strength, and thermal stability because of the better uniform dispersion of ZnO nanoparticles via CGO are presented in Table 3.^[^ ^142^ ^]^ ZnO nanoparticles were previously applied in Table 2.^[^ ^138^ ^]^ The results and the factors are similar, although the flux of No. 4 in Table 2 is less than the flux of No. 7 in Table 3. Better hydrophilicity was reported for both of them.

The production of a membrane containing RGO and SiO_2_ nanomaterials (PVDF/RGO@SiO_2_/PDA) in the removal of 10 mg L^−1^ of the selected dye is presented in the research.^[^ ^135^ ^]^ SiO_2_ between graphene layers is applied to prevent damaging the membrane.^[^ ^135^ ^]^ GO‐based membranes with low water flux can easily be degraded.^[^ ^135^ ^]^ Using SiO_2_ between graphene layers results in high rejection rates and high flux in the separation/filtration of dye‐oil emulsion wastewater, as well as improvements in membrane structure and surface morphology.^[^ ^135^ ^]^ In addition, the deposition of DA with abundant N and O groups on the membrane surface is an effective method for extending a hydrophilic surface in the membrane.^[^ ^135^ ^]^ The highest flux in Table 3 belongs to No. 8, in which the type of membrane is not mentioned. The oil‐water emulsion is applied in this research. Hence oil is often harmful for water membranes, and because of notable flux in comparison with other research in Table 3, the research is interesting and important for oil‐water separation.

Zhu et al.^[^ ^143^ ^]^ only worked with 10 mg L^−1^ Rhodamine B. The TEM test in their research was only performed on nanomaterials (not membrane).^[^ ^143^ ^]^ Zhu et al.^[^ ^143^ ^]^ also reported that by increasing the amount of GO from 0% to 0.9% wt.%, the PVDF/GO/LiCl water flux changed from 484 l/(m^2^h.MPa) to 619 l/(m^2^h.MPa). The synergistic effect of LiCl and GO particles led to the improvement of hydrophilicity and pure water flux for PVDF/GO/LiCl membranes. The optimal dosage of GO was 0.5 wt.% in their study for the M3 membrane. Zhu et al.^[^ ^143^ ^]^ also recommend the use of nanoparticles in membranes to improve their performance in dye recovery and desalination. Dye removal rates of PVDF/GO/LiCl exceeded 80%, and flux recovery ratios of PVDF/GO/LiCl were over 78.2%.^[^ ^143^ ^]^ As seen in Table 3, the first row and two last rows have increased hydrophilicity. In addition, all the NHMs in Table 3 are related to GO. Vice versa, all NHMs in Table 2 are free from GO and related materials.

The following conclusions can be made for Polymeric NHMs produced by combined PI:

- GO and related materials are the most popular materials
- Complicated processes and materials are applied in this section
- Complicated pollutants can be removed but are ineffective in removing salts.
- The oil‐in‐water emulsions containing dye and polar solvents such as ethanol and isopropanol were successfully filtered in this section.
- The high concentration of 500 mg L^−1^ dye was filtered in this part
- Azo, Xanthene, and Thiazine dyes with positive and negative charges were applied

#### 2.2.3 Polymeric NHMs not Produced by PI

**Table** **4** illustrates the summary of findings for non‐photocatalytic polymeric NHMs not produced by PI for dye reduction. The abbreviations in Table 4 are Deep Eutectic Solvents (DESs), Graphitic Carbon Nitride (G‐C_3_N_4_), Silver nanoparticles (Ag NPs), and Cellulose Microgels (CMG), Derivative Thermogravimetry Analysis (DTG), and Polyamide (PA).

**Table 4:** Summary of researchers’ findings for non‐photocatalytic polymeric NHMs which not produced by PI to dyes reduction (continued).

No.	Reference and Year	Membrane Materials	Type of Pollution	Type of Membrane	Method	Analysis	Main Findings	Maximum Flux
10	[145]	DES‐GO/TiO_2_ on the PES membrane	Different negatively charged dye solutions such as Congo Red, Methyl Blue, Evan Blue, and Direct Red	NF, Not mentioned	Sonication, and Filtration	XRD, TEM, Raman spectra, FTIR, Zeta potential, contact angle, SEM, flux	Better performance under ultraviolet (UV) light, Self‐cleaning ultrafast lamellar membrane, 98% dye and 4% Na_2_SO_4_ salt removal, suitable in the dye/salt separation.	2023 l/(m^2^h.MPa)
11	[146]	G‐C_3_N_4_ decorated on RGO with TiO_2_ nanomaterial on the PVDF membrane	Oil, Methylene Blue, Rhodamine B, Methylene Orange	MF, Not mentioned	Electrospun, and Filtration	AFM, TGA, BET, FE‐SEM, XPS, TEM, Pore size, Raman spectra, contact angle, tensile strength, flux, pH, fouling resistance, and filtration cycles	High rejection ratios (40‐100%), excellent stability, low contact angle, increased surface roughness, and surface charges.	11035 l/(m^2^h.MPa)
12	[147]	Ag NPs@CMG^a)^	4‐nitrophenol, Methylene Blue, Methyl Orange, Rhodamine 6G	MF/UF^b)^, Not mentioned	Sol–gel, and Physical mixing/ blending	SEM, TEM, FTIR, XRD, particle size	The straightforwardness, sustainability, and simplicity of the preparation draw application of it in various industrial fields.	‐
13	[61]	GO and Polyelectrolyte Complexes (PECs) (PEI‐modified GO hybrids and PAA on PAN membrane)	Methyl Blue, Methyl Orange, Congo Red	NF, Flat sheet	LbL	SEM, FTIR, Zeta potential, TGA/DTG, AFM, TEM, electrokinetic analyzer, nano‐indenter, flux, mechanical strengthen, water‐solvent kind	GO improves the thermal and hardness stabilities. The retention of Na^+^ and Mg^2+^ were 43.2% and 92.6%, respectively. Retentions: Methyl Orange (87.6%) < Methyl Blue (99.3%) < Congo Red (99.5%).	8.1–12.4 Kg/(m^2^h.MPa)
14	[148]^c)^	PA 66 and PAN)100‐400 nm) on Spunbond‐Meltblown‐Spunbond (SMS) polypropylene nonwoven and carbon	Youha Threne Blue Pffd	MF^a)^, Not mentioned	Electrospun	FE‐SEM, Layer arrangement, Dosage (Electrospun), substrate kind, layer material percent, pressure, time	SMS had finer fibers, therefore, smaller pores, and as a result, had higher flux and efficiency. PAN/PA/PAN membrane on SMS substrate, achieved 86.13% and 99.58% Chemical Oxygen Demand (COD) and turbidity, respectively.	250‐1000 l/(m^2^h.MPa)

^a)^ Polymer microgel;

^b)^ Estimated.

^c)^ Nanohybid according to the Ref. [59] and MF^[^ ^148^ ^]^ is reported in the literature.^[^ ^59^ ^]^

All the polymeric NHMs not produced by PI in Table 4 except the last item were used for complicated pollutants. The type of filtration in this table was MF, NF, and UF. The methods to produce NHMs in this section include sonication, filtration, electrospun, LbL, sol–gel, and physical mixing/blending. There is a big difference between the maximum flux of NF in the first row and MF in the second row of Table 4.

Mehrabi and Aich^[^ ^145^ ^]^ removed initial dye concentration of 10 mg L^−1^ by filtration using the MF membrane manufactured by fixation of DES‐GO/TiO_2_ on the PES substrate, and the final membrane was NF. Although the fabricated membrane had better performance under UV, it was not mentioned as a photocatalytic in the paper; therefore, it was categorized in the non‐photocatalytic polymeric NHMs in Table 4. Although the researchers introduced different nanohybrids such as DES/GO‐Fe_2_O_3_, DES/GOFe_3_O_4_, DES/GO‐Ag, DES/GO‐Au, and DES/GO‐TiO_2_, the nanohybrid membrane of DES‐GO/TiO_2_ on PES was examined.^[^ ^145^ ^]^ Sonication, filtration, electrospun, sol–gel, physical mixing/ blending, and LbL were among the NHM production methods in Table 4.

Venkatesh et al.^[^ ^146^ ^]^ articulated that organic dyes with different charges could be selectively separated. They showed the production of a modified G‐C3N_4_/RGO/TiO_2_ nanofiber PVDF membrane. In the study of Venkatesh et al.,^[^ ^146^ ^]^ TGA showed good thermal stability and even after ten times usage, the membrane did not lose G‐C_3_N_4_/RGO/TiO_2_ nanomaterials. In addition, the adsorptions of the dyes were in the ranges of 18–45 mg g^−1^.^[^ ^146^ ^]^ Increasing surface roughness and surface charges is one output of the study.^[^ ^146^ ^]^ The only oil‐containing research in Table 4 is this work, similar to No. 8 in Table 3. In both pieces of research, the pollutant is Methylene Blue. The second study has higher maximum fluxes than the first one. Both of them were fabricated using two or three different methods. Successful oil‐water emulsion and dye wastewater treatment were observed in both methods.

Han et al.^[^ ^147^ ^]^ showed that silver nanoparticles doped Cellulose Microgels (Ag NPs@CMG) production. They produced Ag nanoparticles with an average size of 8 nm using CMG as a reducer and a supporting matrix.^[^ ^147^ ^]^ In their study, residual alkali acted in the system as a functional accelerant and promoted Ag^+^ reduction using CMG. In the study of Han et al.,^[^ ^147^ ^]^ Ag NPs@CMG reduced three types of organic dyes and 4‐NP. The straightforwardness, sustainability, and simplicity of preparation catalytic high‐efficient membrane, draw its application metal NHMs in various industries.^[^ ^147^ ^]^ Silver nanoparticles were also applied in another research in Table 2,^[^ ^137^ ^]^ which proved effective in pollutant removal.

The researchers illustrated the production of NHMs using polyelectrolyte complexes and GO.^[^ ^61^ ^]^ Using GO improves the thermal, Young's modulus and hardness stability.^[^ ^61^ ^]^ However, more work is still needed to achieve truly molecular‐level dispersion of GO on the substrate surface. They stated that organic–inorganic NHMs are believed to be one of the newest materials for the treatment of dye‐containing wastewater.^[^ ^61^ ^]^ Poly(ethyleneimine)‐modified GO (GO and polyelectrolyte complexes) was assembled onto a hydrolyzed polyacrylonitrile UF membrane, followed by polyacrylic acid according to Scheme one in the study.^[^ ^61^ ^]^ Then the NHMs were subsequently immersed into a polyvinyl alcohol solution and fixed using glutaraldehyde (Scheme one).^[^ ^61^ ^]^ Congo Red retention could reach 99.5% with 8.4 kg/(m^2^h.MPa). The retention of Na^+^ and Mg^2+^ were 43.2% and 92.6%, respectively. The order of retention as listed in Table 4 is Methyl Orange (87.6%) < Methyl Blue (99.3%) < Congo Red (99.5%). The retentions of NaCl (47%) and MgSO_4_ (46%) were obtained in the research.^[^ ^53^ ^]^ Complete NaCl permeation was also reported in the investigation.^[^ ^54^ ^]^ Three items from the five items in Table 4 are carbon‐based. GO can improve thermal and hardness stabilities.^[^ ^61^ ^]^

Mohammadi et al.^[^ ^148^ ^]^ examined two kinds of substrates including SMS and carbon foam for removal of YouhaThrene Blue Pffd (170 mg L^−1^). They mentioned that a reduction in the solution concentration led to finer fibers and smaller pores.^[^ ^148^ ^]^ Mohammadi et al.^[^ ^59^ ^]^ depict the two images with thick and thin fibers that show that the web with lower diameter fibers has higher fluxes because the thickness of thick fibers acts as flux barriers; therefore, thin fibers can provide smaller pores, more fluxes, and better filtration efficiency. Too‐thin fibers cannot guarantee strength. As a result, the Design of Experiments (DOE) methods such as Taguchi and Response Surface Methodology (RSM) can find the optimum point of the average thickness of fibers.^[^ ^3^, ^59^, ^149^, ^150^, ^151^, ^152^ ^]^ Therefore, the application of DOE is an attractive work among the studies related to NHMs used in dye reduction.^[^ ^148^, ^151^ ^]^

The following conclusions can be made regarding polymeric NHMs not produced by PI:

- The type of pollution is complicated except for one research that used the Electrospun method.
- LbL is another technique with electrical charges
- Filtration combined with other processes was mentioned as a process in this section
- Sol–gel and physical mixing/blending is the other processes in this part
- Three of the five pieces of research in this section were produced from carbon‐based material.
- Polymeric NHMs produced by the LbL process cannot remove over 45% Na^+^.
- Sol–gel and physical mixing with various applications in industrial fields were introduced.
- The substrate can affect the NHM's pore size and flux.
- Both surface roughness and surface charges can be increased using nanomaterials.

#### 2.2.4 Ceramic NHMs

**Table** **5** summarizes findings for non‐photocatalytic ceramic NHMs for dye reduction. Both works in Table 5 are NF tubular NHMs produced using the LbL method.

**Table 5:** Summary of findings for non‐photocatalytic ceramic NHMs for dyes reduction (both pieces of research are NF tubular membranes produced using the LbL method).

No.	Reference and Figure	Membrane Materials	Type of Pollution	Analysis	Main Findings	Maximum Flux
15	[153]	Repairing ceramic membranes using polyelectrolyte‐coated nanoparticles	Methyl Blue	SEM, EDX, Pore size, Integrity, Substrate kind, Building blocks (polyelectrolyte molecule weights and structure), flux, pore‐mouth size	High flux and retention, the low cost of substrate repair in the filtration process with retention of 99.1% (0.5 Mpa) for Methyl Blue.	109–314 l/(m^2^h.MPa)
16	[60]	PEI/TiO_2_/PAA, PDDA/PSS, PEI/TiBisLac/PAA on ceramic membranes^a)^	Gongo Red, Methyl Blue	SEM, EDX, Substrate pretreatment, Addition of salt, type (four pairs) and number of layers (1‐20), dry and water‐filled substrates, contact angle, flux	Water pretreatment was beneficial, the addition of NaCl changed the structure. Fast method, easily used in situ for coating large areas.	15–85 Even 3000 Kg/(m^2^h.MPa)

^a)^ TiO_2_ nanomaterials, PAA: Poly (acrylic acid), TiBisLac: Titanium(IV) bis (ammonium lactato) dihydroxide.

Li et al.^[^ ^153^ ^]^ depict inorganic substrates modified with nanoparticles. They conducted notable research by repairing the large defects of macroporous ceramic membranes using polyelectrolyte‐coated nanoparticles. As showed in their study, PDDA‐coated ZrO_2_ nanoparticles (poly(diallyldimethylammonium chloride)‐ ZrO_2_ NPs), PSS‐coated ZrO_2_ nanoparticles (poly(styrene sulfonate)‐coated ZrO_2_ NPs) and PEI‐coated ZrO_2_ nanoparticles (poly(ethyleneimine)‐ ZrO_2_ NPs) were used as polycations and polyanions. Similar to some studies in Table 2, the selected dye is Methyl Blue with 100 mg L^−1^ concentration.^[^ ^153^ ^]^ The organic–inorganic membranes offer high fluxes and retentions.^[^ ^153^ ^]^ According to the study, ZrO_2_ multi‐layers can form a sub‐layer and a dense layer to achieve higher selectivity of the ceramic membrane.^[^ ^153^ ^]^ The structure of building blocks (PE‐coated ZrO_2_ nanoparticles) and the size of substrate pore mouths affect the membrane structure and filtration as shown.^[^ ^153^ ^]^ The low cost of repairing substrate during the filtration process with retention of 99.1% (0.5 MPa) for methyl blue played an important role in the hetero‐structure results, which resulted in dye removal.^[^ ^153^ ^]^

The studies of Li et al.^[^ ^153^ ^]^ and Tang et al.^[^ ^60^ ^]^ are similar to each other from the views of production method (LbL) and type of membranes (ceramic). Tang et al.^[^ ^60^ ^]^ examined different membranes according to Table 5 and expressed that substrate pretreatment using water filling was beneficial to forming multi‐layers on the surface and changing morphology by decreasing the polyelectrolyte penetration rate into the pores of the substrate. The addition of NaCl to the polyelectrolyte solution could change the multilayer structure. Therefore, the dye rejection performance can be adjusted using water or salt pretreatment and other pretreatments. The five layers of the PEI/PAA membrane had a rejection of > 99% for Gongo Red, Methyl Blue, and Acid Fuchsin organic dyes (100 mg L^−1^) and a flux of 10 Kg/(m^2^h). This rapid NHM production method accomplished by spraying on vertically held tubular Al_2_O_3_ substrates can be easily used for different polymeric multi‐layers in situ for coating large areas.^[^ ^60^ ^]^

Like polymeric NHM produced using the LbL process, the type of filtration is NF for both ceramic NHMs. Carbon‐based materials were not applied as nanomaterials in this part, although they were applied in polymeric NHMs. The maximum flux in LbL polymeric NHM is close to ceramic NHM. As a conclusion for the ceramic NHMs section, LbL is a fast method easily used in situ for coating large areas. Water pretreatment was beneficial. High flux and retention, and low cost for substrate repair in the filtration process with high retention are among the other benefits of this method. The number of layers can be as high as 20.

#### 2.2.5 Non‐Polymeric and Non‐Ceramic NHMs

**Figure 8:**
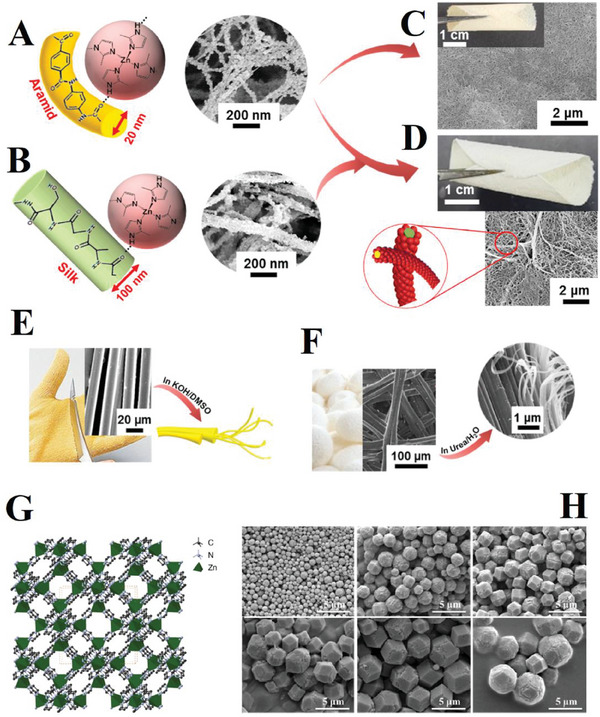
Web‐like NHMs production by changing Aramid Nanofibrils (ANFs) and Silk Microfibrils (SilkMFs) using Zeolitic Imidazolate Framework‐8 (ZIF‐8).^[^ ^58^ ^]^ ZIF‐8 crystals (13–42 nm) coating on ANFs and SilkMFs is also illustrated by red circles. A) Loading ZIF‐8 on ANFs, B) Loading ZIF‐8 on SilkMFs, C) ANFs and ZIF‐8 membrane, D) ANFs, SilkMFs, and ZIF‐8 web‐like NHM. E) Exfoliation into ≈20 nm‐thick ANFs. F) Exfoliation into ≈100 nm‐thick SilkMFs. Reprinted with permission. G) ZIF structure.^[^ ^154^ ^]^ Reprinted with permission. (H) ZIF‐8 with different sizes.^[^ ^155^ ^]^ Reprinted with permission from the research.^[^ ^155^ ^]^ 2018 American Chemical Society.

**Table** **6** shows the summary of researcher findings for non‐photocatalytic, non‐polymeric, and non‐ceramic NHMs for dye reduction. **Figure** **8** in the present work is mentioned in Table 6 for better conceptualization.

**Table 6:** Summary of findings for non‐photocatalytic, non‐polymeric, and non‐ceramic NHMs for dyes reduction (MF is estimated for Ghani et al.^[^ ^150^ ^]^ and reported for Lv et al.^[^ ^58^ ^]^ Ghani et al.^[^ ^150^ ^]^ used NHM^[^ ^151^ ^]^).

No.	Reference and Figure	Membrane Materials	Type of Pollution	Method	Analysis	Main Findings
17	[58] Figure 8, ^a)^	Nanofibers. ZIF‐8 coating on ANFs (20 nm) and SilkMFs (100 nm (	Malachite Green, Coomassie Brilliant Blue, Congo Red, Methyl Violet, Methylene Blue, Rhodamine 6G, Rhodamine B	Filtration followed by solvent exchange and freeze‐drying	TGA, FTIR, XRD, FE‐SEM, BET, diameter distributions, reuse cycle	The large aspect ratio and flexibility. Large solvent flux. High rejection ratio (≥99%). Membranes with hierarchical microstructures and possible industrial production.
18	[151]	Alginate/Polyethylene oxide (Alg/PEO) with 142 nm diameter	Acid Red 14, Basic Blue 41	Electrospun	SEM, FTIR, time, pH, dosage, dye concentration, isotherms, voltage, distance, diffusion	Alg is suitable for adsorption of cationic/anionic dyes because of notable adsorption sites on the nanofiber surface, large ratios of surface to volume, surface charge adjustment via the solution pH, and low weight.

^a)^ Maximum flux: 3900 L/(m^2^h.MPa)

According to Figure 8, Lv et al.^[^ ^58^ ^]^ studied web‐like hybrid membranes with 43 wt.% of ZIF‐8, 19 wt.% of SilkMFs, 38 wt.% of ANFs, and a thickness of 120 µm for wastewater treatment. G and H parts in Figure 8 were added to the other selected images of the Lv et al.^[^ ^58^ ^]^ paper for better illustration. The thicknesses of the fibrils are mentioned in Table 6. Fibrous membranes are fabricated using loading engineering Metal‐Organic‐Frameworks (MOFs) by adsorption and filtration mechanisms on the porous substrate of electrospun nanofibers and fabric fibers via passing through the substrate's meso or macro pores.^[^ ^58^ ^]^

The low rejection ratios can be attributed to insufficient MOF loading and time, the NHM also could be reused at least five cycles without an important performance compromise and just a 2% reduction in rejection ratio after the fifth cycle.^[^ ^58^ ^]^ Exfoliation of the fibers was also accomplished.^[^ ^58^ ^]^ In their study, the initial support was a commercial membrane with a pores size of 220 nm. Filtration was followed by solvent exchange using tertiary butanol and freeze‐drying. In addition, the concentration of dyes was 50 mg L^−1^.^[^ ^58^ ^]^

Besides the MOF membranes invented by the Yaghi Research Group in 1995, the Covalent Organic Frameworks (COFs) membrane introduced in 2005 by the Cote Study Group is applied for dye elimination.^[^ ^156^, ^157^ ^]^ Porous aromatic frameworks (PAFs) are also mentioned for pollutant removal.^[^ ^158^ ^]^

Ghani et al.^[^ ^150^ ^]^ illustrated alginate‐based nanofibers production and adsorption of anionic and cationic dyes. They accomplished three optimization steps to obtain the desired membrane morphology, Alg content, and lowest possible diameter of nanofibers with initial dye concentrations of 50 mg L^−1^. Alg/PEO nanofibers with an 80:20 ratio were synthesized. The maximum performance at pH of 9 and 1 was reported at 71% and 93% for Basic Blue 41 and Acid Red 14, respectively.^[^ ^150^ ^]^

None of the membranes in this section were produced from carbon‐based materials, and thus, are all fiber‐based. The last item in Table 4 can also be mentioned in this part because only the substrate is made of carbon. The large aspect ratio and flexibility, large solvent flux, high rejection ratio, hierarchical microstructures, and possible industrial production are other properties that can be mentioned for fiber‐based NHMs.

### 2.3 Photocatalytic NHMs

The summary of findings for photocatalytic NHMs for dye reduction is described in **Table** **7**. More works are available in photocatalytic NHMs for dyes reduction,^[^ ^65^, ^164^ ^]^ but after evaluation, the word nanohybrid was not found in the referred paper,^[^ ^164^ ^]^ therefore to avoid any mistake, the papers lacking the word NHM are not mentioned in Table 7. Some abbreviations in the membrane's analysis or nanomaterials of Table 7 are Barrett Joyner Halenda (BJH), Diffuse Reflectance Spectrometry (DRS), Surface Free Energy (SFE), Liquid Chromatography–Mass Spectrometry (LC–MS), Powder X‐Ray Diffraction (PXRD), Ultraviolet‐visible (UV–vis), High Angle Annular Dark Field Detector‐Scanning Transmission Electron Microscopy (HAADF–STEM), Inductively Coupled Plasma‐Optical Emission Spectrometry (ICP‐OES), Ultra Performance Liquid Chromatography‐High Resolution Mass Spectrometry (U‐HPLC‐HRMS), and Cyclic voltammetry (CV). Strong Reactive Oxygen Species (ROS) caused dye removal.^[^ ^160^ ^]^ The results recommend NHMs for wastewater treatment, supercapacitors, desalination, and biosensors.^[^ ^160^ ^]^ An electrode can be used for multi‐implementation.^[^ ^161^ ^]^

**Table 7:** Summary of findings for photocatalytic NHMs for dyes reduction.

Reference	Membrane materials	Type of pollution	Method	Analysis	Main findings
[159]	Spinel NiFe_2_O_4_/GO on the PVDF membrane	Remazol Red RB‐133 (real industrial wastewater)	Sonication, and PI	SEM, EDX, BET‐BJH, DRS, Energy gap (Tauc plots), AFM, FTIR, XRD, porosity, PSD, with and without UV, contact angle, tensile strength and elongation, SFE, water uptake ability, COD, LC‐MS, kinetic, mechanisms, scavengers’ effects, stability, antifouling, resistances	The insignificant effect of photolysis on overall dye removal is (maximum 15% removal), Added GO nanoparticle to the photocatalyst of NiFe_2_O_4_/GO to prevent the fast rate of electron/hole recombination in NiFe_2_O_4_ photocatalyst, better stability, 83% of COD and 92% of the dye removed, better flux under UV, with the maximum flux of 60 l/(m^2^h.MPa) approximately
[152]	PMOF@PSF_30%_ PMOFs: POMs encapsulated in MOFs Main materials: Phosphotungstic acid hydrate (POM: H_3_PW_12_O_40_⋅xH_2_O), 2‐methylimidazole, and Cobalt nitrate hexahydrate (Co(NO_3_)_2_⋅6H_2_O), PSF membrane	Methylene Blue	Sonication, Physical mixing/ blending, and heating^a)^	FTIR, PXRD, TGA, SEM, TEM, the ratio of material^b)^, UV–vis	RSM was applied, and the optimal results were 98% of dye removal (0.15 g for concentration, 70 °C for temperature, with an 80 min reaction time), great reusability after 10 cycles, evaluation of three parameters of photocatalyst loading (0.05‐0.15 g), degradation temperature (30–70 °C), and degradation time (40–120 min)
[160]	NiO nanoparticles and nano chitosan (Cs)	Rhodamine B and Methylene Blue	Physical mixing/ blending	XRD, SEM, FTIR, Zeta potential, Energy gap, EDX, TEM, Cytotoxicity, CV, polarization resistance, Tafel curve, thermal performance	Rhodamine B: 80%, 37%, and 27%, for Cs@NiO, Cs@NiO NHM, and NiO, respectively. Methylene Blue: 86%, 77%, and 29% for Cs@NiO, Cs@NiO NHM, and NiO NPs, respectively.
[161]	Y_2_O_3_ and GO/Carboxymethyl cellulose (CMC): GO@Y_2_O_3_, CMC@Y_2_O_3_, GO@CMC.Y_2_O_3_	Methylene Blue	Precipitation, and Ultrasonication	FTIR, Zeta potential, SEM, EDX, TEM, XRD, CV, polarization resistance, Tafel curve	Methylene Blue removed 52% without help from other catalysts such as (H_2_O_2_).
[162]	Molybdenum Disulfide (MoS_2_) and TiO_2_ nanohybrids on PAN	Rhodamine B, Methyl Orange, Rhodamine 6G, Malachite Green, Methylene Blue	Electrospun, and filtration	SEM, TEM, XRD, Raman spectra, XPS, mechanism of photocatalysis	MoS_2_ nanosheets ensured sufficient active sites. TiO_2_ nanotubes could act as spacers to isolate MoS_2_ and can increase the active sites and surface area.
[163]	Poly(m‐phenylene isophthalamide) (PMIA) enhanced by immobilization of GO, ZnO, and Ag nanoparticles	Methylene Blue and Cr (VI)	Physical mixing/ blending, and PI	XPS, FTIR, EDX, AFM, TEM, XRD, SEM, flux and rejection, permeability, tensile stress, contact angle, stability, fouling resistance, filtration cycles	Satisfactory self‐cleaning. A negative charge of Hydrophilic GO‐ZnO‐Ag improved permeability. Cr (VI) and Methylene Blue were removed at 71% and 78.1%, respectively.
[55]	Phosphorene nanoparticles and PSF and Sulfonated Poly Ether Ether Ketone (SPEEK)	Methylene Blue and Protein	Physical mixing/ blending, and PI	TEM, AFM, Zeta potential, HAADF–STEM, FTIR, XPS, SEM, pore size, contact angle, CP‐OES, flux, stability, toxicity, leaching, pH, phosphorene distribution	Dispersed phosphorene throughout the membrane matrix led to a 70% reduction in dye fouling after filtration. Phosphorene membranes have rougher surfaces. Less than 1% phosphorene leaching.
[105]	GO, ZnO nanoparticles, and PES	Trace Organic Compounds (TOrCs) and Brilliant Black	PI (Double‐casting)	TEM, Raman spectra, FE‐SEM, AFM, Zeta potential, DLS, U‐HPLC‐HRMS, contact angle, porosity, tensile strengths, the pressure measured, conductivity, flux	High fluxes, rejection, and photocatalytic properties at low (0.4 MPa) pressures: it can be applied in developing countries especially in rural areas with limited water without other treatment processes.

^a)^ It was similar to sol–gel. Finally, PMOF@PSF was annealed at 150 °C;

^b)^ 5%, 10%, 20%, 30%, and 40% of the filler.

Kusworo et al.^[^ ^159^ ^]^ removed ≈154 mg L^−1^ Remazol red RB‐133 dye using PVDF/NiFe_2_O_4_/GO membrane. Numerous analyses were accomplished for the applications of spinel NiFe_2_O_4_/GO on the PVDF membrane.^[^ ^159^ ^]^ Other membranes including neat PVDF, PVDF/GO, and PVDF/NiFe_2_O_4_ were also tested for comparison.^[^ ^159^ ^]^ Better stability is also reported in comparison with other membranes.^[^ ^159^ ^]^ Although UV was good for pollutant removal and flux, the impact was not significant.^[^ ^159^ ^]^

Hassan et al.^[^ ^152^ ^]^ applied a three‐level Box‐Behnken Design (BBD) in the research for eliminating 40 mg L^−1^ solutions of Methylene Blue in 50 mL of water using the PMOF@PSF membrane. The membrane comprises POMs encapsulated in MOFs supported by PSF.^[^ ^152^ ^]^ The research focused on mixed matrix membrane (MMM) fabrication with a POM‐based metal‐organic framework (PMOF) as a nanohybrid filler in PSF to obtain PMOF@PSF.^[^ ^152^ ^]^ The membrane proved successful in dye removal.^[^ ^152^ ^]^

As mentioned in Table 7, Zhang et al.^[^ ^162^ ^]^ worked with a high initial dye concentration of 5000 mg L^−1^ of different dyes. They expressed that MoS_2_ nanosheets are excellent active materials with proper photodegradation performance to fabricate MoS_2_‐TiO_2_@PAN membranes to remove organic dyes.^[^ ^162^ ^]^ The large surface area of MoS_2_ nanosheets ensured sufficient active sites.^[^ ^162^ ^]^ In addition, TiO_2_ nanotubes could act as spacers to isolate MoS_2_ and can increase the active sites and surface area.^[^ ^162^ ^]^

According to Xie et al.,^[^ ^163^ ^]^ the designed membrane improved Methylene Blue and Cr (VI) photocatalytic degradation, although the initial concentration (20 mg L^−1^) similar to the other research^[^ ^55^ ^]^ with 10 mg L^−1^, was too low and the membranes were not examined in high concentrations. Mahlangu et al.^[^ ^105^ ^]^ used just 2–4 mg L^−1^ Brilliant Black as the lowest value in Table 7. Satisfactory self‐cleaning in the photocatalytic process was observed by Xie et al.^[^ ^163^ ^]^ Degradation efficiencies of 71% and 78.1% were reported for Cr (VI) and Methylene Blue, respectively.^[^ ^163^ ^]^ Eke et al.^[^ ^55^ ^]^ introduced phosphorene was introduced as a substance with inherent photocatalytic properties and electrical conductivity. Distribution of phosphorene was also assessed on the membrane.^[^ ^55^ ^]^ Eke et al.^[^ ^55^ ^]^ measured a flux of 107 L/(m^2^h) in 0.206 MPa. A high flux of 519.4 L/(m^2^h.MPa) was also reported.^[^ ^55^ ^]^ In addition, minimal released phosphorene from the membranes (less than 1% phosphorene leaching) was reported, which caused relatively low toxicity in its free form.^[^ ^55^ ^]^ The type of membrane was NF/UF.^[^ ^55^ ^]^

According to the high fluxes, rejection, and photocatalytic properties at low (0.4 MPa) pressures, the NHM in Table 7 is applied in developing countries especially in rural areas with limited water without other treatment processes.^[^ ^105^ ^]^ According to Mahlangu et al.,^[^ ^105^ ^]^ GO‐ZnO/PES photocatalytic membranes are suitable for degrading dyes. GO‐ZnO increases hydrophilicity, flux, effluents containing salts, resistance to fouling by organics, photodegradation, and rejection. TOrCs were rejected because of membrane hydrophilicity properties.^[^ ^105^ ^]^ MF/UF can be also estimated as a type of membrane in this study.^[^ ^105^ ^]^

According to Tables 2, 3, 4, similar to non‐photocatalytic NHMs, polymeric membranes are the most famous photocatalytic NHMs applied in dye removal.^[^ ^55^, ^105^, ^162^, ^163^ ^]^ Organic‐inorganic materials were used in 50% of the reviewed photocatalytic NHM studies.^[^ ^55^, ^160^, ^162^ ^]^ In addition, 50% of the materials of photocatalytic NHM studies were carbon‐based.^[^ ^105^, ^163^ ^]^ MF/UF,^[^ ^105^ ^]^ UF,^[^ ^152^ ^]^ and UF/NF^[^ ^55^ ^]^ were applied in Table 7; in other researches the pore sizes were not mentioned. PI as one method of photocatalytic NHM production was applied in 50% of the cases presented in Table 7.^[^ ^55^, ^105^, ^163^ ^]^ From the view of publication time, 86% of the photocatalytic NHMs papers in Table 7 were published after 2018, and only one research was published before 2018.^[^ ^55^, ^105^, ^160^, ^161^, ^162^, ^163^ ^]^

The following conclusions can be drawn regarding photocatalytic NHMs:

- The pollutants studied in this section varied greatly, ranging from single dyes to mixtures of dyes and other pollutants.
- Different methods were employed for membrane production, including sonication, PI, physical mixing/blending, heating, precipitation, ultrasonication, electrospinning, and filtration.
- The concentration of dyes used ranged from 2 to 500 mg L^−1^.
- The most commonly used material for membrane fabrication was GO, with a frequency of 50%.
- With the exception of one, all the membranes discussed in this section were metallic.
- High dye removal rates, exceeding 80%, were observed particularly at high dye concentrations (500 mg L^−1^), indicating effective degradation of the selected dyes.

## 3 Conclusion and Recommendation for Further Works

NHM production as an advanced material with application in dye removal from 2018 up to now, improved three times during the past five years. Using membranes for dye separation is more favorable than other treatment methods because the dye can be recycled in the industry. Although photocatalytic NHMs degrade dyes instead of completely recycling them, they were assessed in the current research as a type of NHM. Ineffective performance, fouling problems, and finding a solution for repairing expensive available membranes resulted in the production of NHMs. The application of NHMs cannot promise better performance in comparison with other types of membranes. NHMs' pertaining challenges and problems include 1) not being effective in reducing small‐size ions,^[^ ^52^, ^53^, ^54^ ^]^ 2) aggregation and leaching,^[^ ^55^ ^]^ 3) stability of the membrane or materials,^[^ ^55^, ^56^, ^57^ ^]^ 4) being expensive,^[^ ^10^ ^]^ and 5) needing high technique.^[^ ^58^ ^]^

NF is the predominant filter type fabricated among NHMs for dye removal. Though not that different from MF and UF. PI is the most famous method and carbon is the most popular material in NHM production for decolorization. Among carbon‐based materials, GO and its family have numerous applications as an NHM material, including the porous structure of GO that offers high fluxes. Metal/metal oxides have stable structures and because of their charges and hydrophilic properties that result in antifouling properties are popular for NHM. CNTs with robust tubular structure are a novel form of stable carbon, which can be applied in NHMs for dye reduction. Polymers and fibers are inexpensive and lightweight. According to the results, ceramic membranes are expensive and strong; therefore, inexpensive and simple repair of ceramic membranes was another attractive field in NHMs production.

Suggestions for further research are:

- NHMs can be used in the recycling line of the textile industry, but the dye concentration is usually as high as 200–500 mg L^−1^. Most studies have investigated the initial dye concentration of 100 mg L^−1^. Therefore, further studies closer to real‐world conditions are needed.
- Textile industries’ effluents are salty, so like some research evaluated in this study, more works can overcome this problem, or at least propose substituting raw materials such as salts in the textile industry.
- Application and synthesis of more NHMs for decolorization, especially from other/new raw materials or NHMs produced from methods other than what was mentioned in this paper.
- Comparison of the cost‐benefits of several NHMs for textile dye reduction is necessary. Techno‐economical evaluation with the help of artificial intelligence can show the practicality of NHMs’ synthesis.
- DOE can also investigate the importance of each parameter in NHM manufacturing. The application of RSM as a prominent DOE method received considerable attention this year.

## Conflict of Interest

The authors declare no conflict of interest.

## Supporting information

Supporting Information
