# Supplementary material for: Advances in Nanohybrid Membranes for Dye Reduction: A Comprehensive Review
Source: Glob Chall. 2023 Dec 21;8(1):2300052. doi: 10.1002/gch2.202300052 (PMC10784202; doi:10.1002/gch2.202300052)
Supplement: Supplementary file 1 — Supporting Information [file GCH2-8-2300052-s001.pdf]

Global Challenges

Open Access

Supporting Information

for *Global Challenges.*, DOI 10.1002/gch2.202300052

Advances in Nanohybrid Membranes for Dye Reduction: A Comprehensive Review

*Mahsa Taheri**

Supporting Information

Nanohybrid based membrane for dyes reduction: A review

Mahsa Taheri

Civil and Environmental Engineering Department, Amirkabir University of Technology (AUT), Hafez Ave., Tehran, 15875-4413, Iran

E-mail: mahsa_taheri88@yahoo.com

[*] Corresponding Author. Tel.: +9821-64543000; Fax: +9821-66414213

List of table(s):

Table S1 Categories of non-photocatalytic NHMs for dyes reduction

Table S1 Categories of non-photocatalytic NHMs for dyes reduction

Parameters		Reference (No in Tables 2-6)	Percent
Type of materials	Carbon-based*	3, 5, 6, 7, 8, 9, 10, 11, 13	50%
	Non carbon-based**	1, 2, 4, 12, 14, 15, 16, 17, 18	50%
Size of pores	MF	2, 11, 14, 17, 18	27.78%
	MF/UF	12	5.55%
	UF	3, 4, 6, 9	22.22%
	UF/NF	1	5.55%
	NF	5, 7, 10, 13, 15, 16	33.33%
	Not mentioned	8	5.55%
Shape of membrane	Flat sheet	13	5.55%
	Hollow fiber	5, 6, 7	16.67%
	Tubular	15, 16	11.11%
	Not mentioned	1, 2, 3, 4, 8, 9, 10, 11, 12, 14, 17, 18	66.67%
Method of production	PI and other processes	1, 2, 3, 4, 5, 6, 7, 8, 9	50%
	Other processes	10, 11, 12, 13, 14, 15, 16, 17, 18	50%
Year of publication	After 2018	1, 2, 3, 4, 5, 6, 7, 8, 10, 11, 14, 17	66.67%
	Before 2018	9, 12, 13, 15, 16, 18	33.33%

* Including carbon-organic, carbon-inorganic, and carbon-organic-inorganic

** Including organic, inorganic, and organic-inorganic
